# Stereotactic radiotherapy for brain metastases: indications, dose fractionation, technological innovations, and evolving combination strategies – a comprehensive review

**DOI:** 10.3389/fonc.2026.1821752

**Published:** 2026-04-23

**Authors:** Hu Chen, Shuai Li, Qiuyu Yang, Fangzheng Zhou

**Affiliations:** 1 Department of Radiation Oncology, Shenzhen Luohu Hospital of Traditional Chinese Medicine(Shanghai University of Traditional Chinese Medicine, Shenzhen Hospital), Shenzhen, China; 2 Department of Oncology and Hematology, Shenzhen Luohu Hospital of Traditional Chinese Medicine(Shanghai University of Traditional Chinese Medicine, Shenzhen Hospital), Shenzhen, China; 3 Department of Radiation Oncology, Shenzhen Luohu Hospital Group Luohu People’s Hospital (Third Affiliated Hospital of Shenzhen University), Shenzhen, China

**Keywords:** advance, brain metastases, combined modality therapy, stereotactic radiotherapy, stereotactic radiotherapy equipment

## Abstract

The incidence of brain metastases in cancer patients is increasing due to improved survival from systemic therapies and advancements in diagnostic imaging. Radiotherapy remains a cornerstone in the management of brain metastases. Among the various techniques, stereotactic radiotherapy (SRT) is widely adopted in multidisciplinary treatment owing to its high precision, rapid dose fall-off, high rates of local control, and superior preservation of neurocognitive function. In recent years, significant progress has been made in several areas of SRT, including technological advancements in equipment and radiation quality, its combination with surgery (either pre- or post-operatively), integration with systemic therapy (such as targeted and immunotherapy), and the exploration of new indications (e.g., more than 4 brain metastases, small cell lung cancer). Furthermore, optimization of dose fractionation regimens—including fractionated SRT, low-dose radiotherapy, exploration of maximum tolerated doses, and individualized ultrafractionated adaptive radiotherapy—has been actively investigated. This review synthesizes the evidence from classical literature and recent clinical studies to outline the current landscape and future directions of SRT for brain metastases.

## 1 Introduction

**Figure 1:**
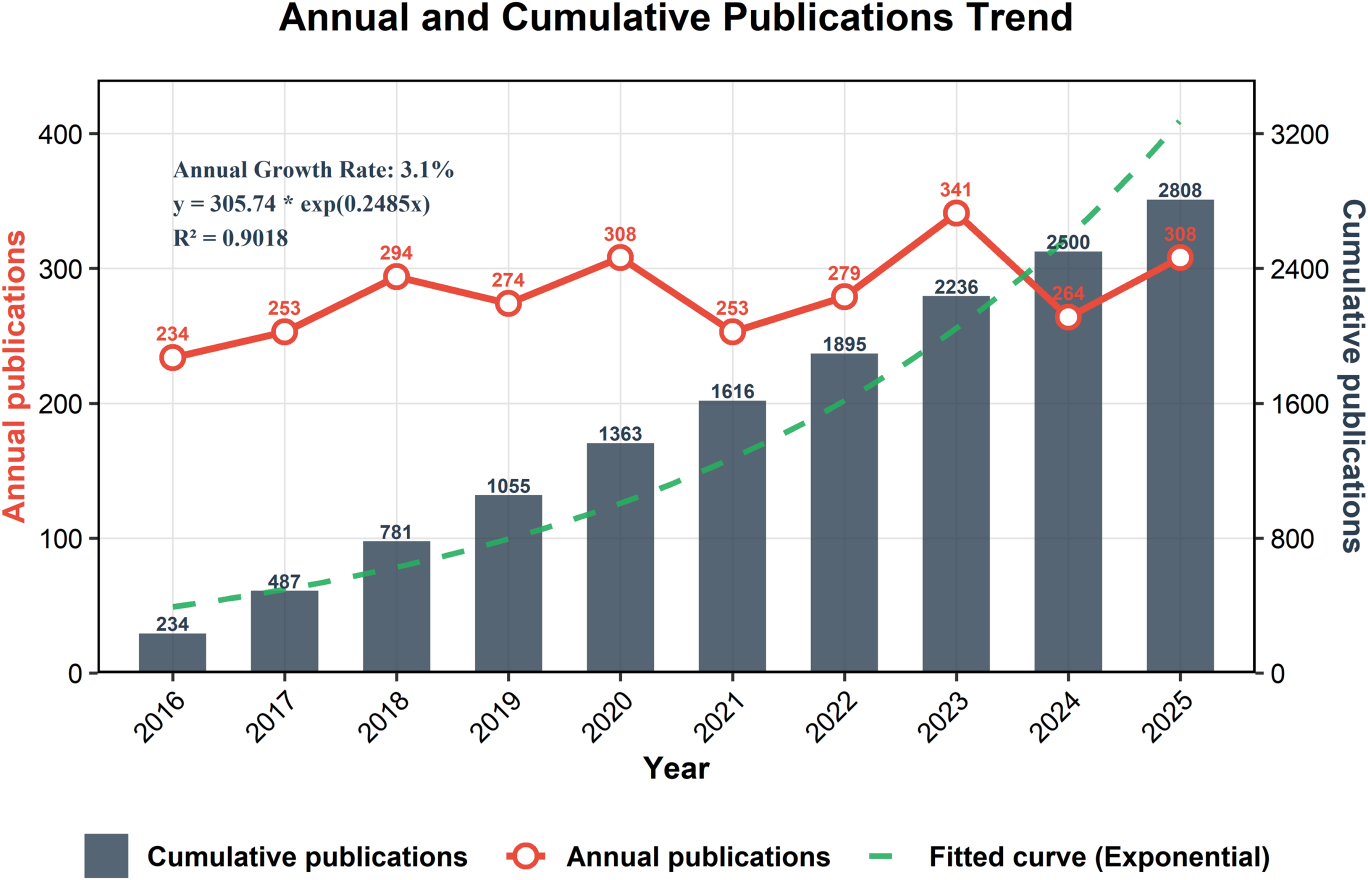
Records were identified through Web of Science Core Collection search for articles published between January 1, 2016, and November 31, 2025, with titles containing terms related to brain metastases and stereotactic radiotherapy. A total of 2,808 articles were included in the final analysis. In the figure, annual publications are depicted by the red line, cumulative publications volume by the gray bars, and the fitted curve for cumulative publications by the green dotted line, with high goodness-of-fit (R^2^ > 90%).

**Figure 2:**
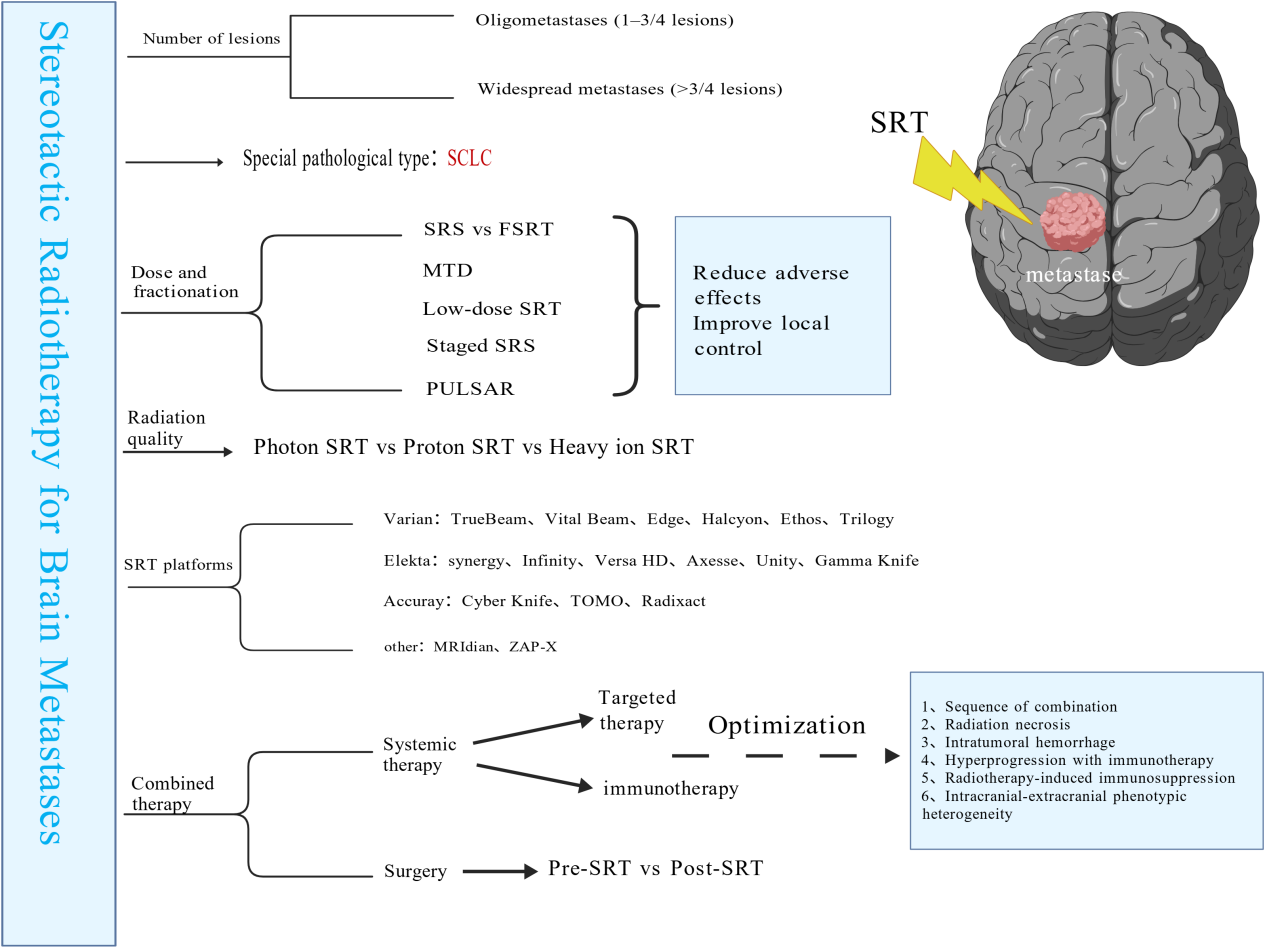
Advances in Stereotactic Radiotherapy for Brain Metastases. This figure outlines the structure of the review and summarizes the advances and optimization strategies in stereotactic radiotherapy for brain metastases. Abbreviations: SRT, stereotactic radiotherapy; SRS, stereotactic radiosurgery; FSRT, fractionated stereotactic radiotherapy; SCLC, small cell lung carcinoma. Created with BioGDP.com. See Jiang, Shuai et al. (2025, DOI: 10.1093/nar/gkae973) for details.

Brain metastases (BM) represent the most prevalent type of intracranial tumor. In recent years, stereotactic radiosurgery (SRS) has seen increasing adoption in the radiotherapy of brain metastases, owing to its advantages of high targeting accuracy and highly conformal dose distribution. A literature search in the Web of Science Core Collection reveals that over the past decade (January 1, 2016, to January 1, 2026), annual publication output in this field has shown a gradual yet fluctuating upward trend (**Figure 1**). This pattern suggests sustained and unconcluded research interest, highlighting the area’s ongoing potential. This review synthesizes prior literature to outline the latest trends and advancements in stereotactic radiotherapy for brain metastases (the overall framework of this review is presented in **Figure 2**).

## 2 Stereotactic radiotherapy for brain metastases: key aspects and evolving strategies

### 2.1 Stereotactic radiotherapy for oligometastatic and multiple brain metastases

#### 2.1.1 SRT in oligometastatic brain disease

Supported by evidence from several large randomized controlled trials (RCTs) (1–3), SRT is widely used in the clinical management of oligometastatic brain disease. The JROSG 99–1 study (1), which compared WBRT + SRS versus SRS alone, demonstrated that the addition of WBRT did not improve overall survival (OS), although it enhanced intracranial control. Two other major Phase III RCTs (ALLIANCE N0574 (2)and NCCTG N107C/CEC.3 (3)) showed that compared to SRS alone, the combination of WBRT with SRS—whether for intact brain metastases or surgical cavities—led to worse neurocognitive function (NCF, a set of higher-order cognitive processes encompassing memory, learning, processing speed, executive function, and verbal fluency, commonly assessed with tools such as the Hopkins Verbal Learning Test-Revised, Trail Making Test, and Controlled Oral Word Association Test (4)) without providing an OS benefit. Furthermore, advances in systemic therapy have established that SRT combined with systemic agents can also maintain high levels of intracranial control (5–7), further diminishing the value of WBRT. Additionally, intracranial progression can often be managed with salvage radiotherapy (either WBRT or further SRT) (8, 9). Consequently, for oligometastatic brain metastases, SRT is a key component of standard treatment per guideline and consensus recommendations (10, 11). However, several aspects still require optimization:

##### 2.1.1.1 Histological type

The aforementioned RCTs comparing SRS and WBRT enrolled patients with mixed histologies, with a predominant representation of non−small cell lung cancer brain metastases (NSCLC−BM) (2, 3). Therefore, when extrapolating their conclusions, decisions must take into account the specific histologic type (especially for rare primary tumors that metastasize to the brain), as well as natural history, CNS tropism, radiosensitivity, and response to systemic therapy.

Melanoma brain metastases (MBM) serve as a typical example. They have a higher lifetime risk of brain dissemination compared to other histologies (12) and are relatively radioresistant to conventional fractionation – a characteristic that may explain why adjuvant WBRT after local therapy (surgery or SRS) failed to improve intracranial distant control(IDC) in a Phase III trial (13). On the other hand, targeted and immunotherapies have demonstrated favorable intracranial activity in MBM, and their combination with SRT improves IDC (6, 7), pointing to a future treatment paradigm. Other radioresistant histologies include renal cell carcinoma, sarcoma, and gastrointestinal cancers; their incidence of brain metastases is low, and consequently prognostic data are scarce (14, 15). A systematic review of SRT for sarcoma brain metastases, comprising nine retrospective studies (272 patients, 745 lesions), reported 1−year local control rates ranging from 0 to 78.3% and 1−year OS from 29% to 37.3% (14). Future multi−institutional collaborative efforts are needed to accumulate more cases and better characterize outcomes for these rare tumor types.

Breast cancer brain metastases (BCBM) appear to have a more favorable prognosis with SRT. Large retrospective studies suggest an OS benefit of SRS over WBRT (16). In a study using 5−fraction FSRT (22.5–35 Gy), doses above 30 Gy were associated with better local control in HER2−positive BCBM compared to other molecular subtypes (*P* = 0.0147) (17). Moreover, an interim analysis of the prospective SAFER study found that BCBM seem more radiosensitive than other histologies: regardless of the dose regimen (16–24 Gy/1 fx or 21–24 Gy/3 fx), the predicted 9−month local control was >90%. For non−breast histologies, a BED_10_ ≥48 Gy was needed to achieve >90% 9−month local control. However, this was an unplanned analysis and requires validation. The SAFER study is expected to enroll 375 patients and will run until 2028 to include 2−year follow−up for all enrolled patients (18).

Taken together, histology− and even molecular subtype−guided research remains necessary to enable individualized radiotherapy decisions based on intrinsic tumor biology. For instance, a tumor control probability model found that while a single fraction of 18 Gy achieves excellent local control for lesions ≤20 mm in most histologies, melanoma brain metastases require a single−fraction dose of 23.4 Gy to achieve >90% local control (19).

##### 2.1.1.2 Lack of contemporary systemic therapy

recent years have witnessed advances in the systemic therapy of brain metastases, particularly driven by molecular profiling and histology-specific treatments (20, 21). However, the patient data in the aforementioned RCTs were collected during earlier eras (JROSG 99-1 (1): 1999-2003; ALLIANCE N0574 (2): 2002-2013; NCCTG N107C/CEC.3 (3): 2011-2015). Combining radiotherapy with modern systemic therapies can improve patient outcomes, as evidenced by robust data in MBM, BCBM, and NSCLC-BM (6, 7, 22–24). For example, A multivariable analysis in EGFR/ALK−positive NSCLC-BM (mostly treated with osimertinib) demonstrated that adding SRS to TKI therapy significantly reduced the risk of intracranial progression (*P* = 0.033), though it did not improve OS (*P* = 0.5) (5). In contrast, The Phase III HYBRID study, which compared SRT and WBRT in EGFR-mutant NSCLC-BM patients receiving first-generation TKIs, was limited by its single-center design, the concurrent publication of studies favoring SRS over WBRT (2, 3), and declining use of first-generation TKIs after osimertinib became standard care (25). Consequently, poor accrual led to underpowered results that cannot support definitive conclusions (26). There is a clear need for well-designed studies based on molecular/biological stratification to truly implement the principles of precision and personalized medicine.

#### 2.1.2 The controversy of SRT for ≥4 brain metastases

A higher number of brain metastases is associated with an increased likelihood of micrometastases and greater subsequent costs of salvage radiotherapy (27). Furthermore, such patients often present with a lower DS-GPA score, and competing risk events for OS may occur before the NCF benefits of SRT can be realized, potentially offsetting its advantages (8). Despite these considerations, numerous studies have evaluated the role of SRT in patients with multiple brain metastases (28–31).

The landmark JLGK0901 study demonstrated non−inferiority of SRS for 5–10 versus 2–4 brain metastases in OS, though patients with a single metastasis fared better (28); its updated follow−up subsequently confirmed the non−inferiority in NCF and SRS−related adverse events (29). However, direct head-to-head comparisons between SRS and WBRT specifically in the setting of multiple (≥4) brain metastases warrant further attention, particularly as WBRT techniques have advanced. The NRG CC001 trial established that hippocampal-avoidance WBRT (HA-WBRT) combined with memantine better preserves NCF and quality of life compared to conventional WBRT (4). Meanwhile, retrospective studies have suggested that WBRT(with or without HA) plus a simultaneous integrated boost (SIB) may achieve both local control and reduced distant intracranial failure, representing a viable alternative (32, 33).

Given these developments, a key clinical question arises: in patients with multiple(≥4) brain metastases, does SRT maintain its previously established NCF advantage over WBRT when compared against modern WBRT incorporating NCF-protective strategies and, further, with SIB techniques?

Early evidence supporting SRT in this context came from a small Phase III trial of 72 patients with 4–15 brain metastases, which found no significant differences in intracranial local control(ILC), IDC or OS between SRS and WBRT; among the 31 patients evaluable for NCF, SRS maintained an advantage at 4 months even though 62% of the WBRT group received memantine (30).

More recently, a multicenter Phase III trial (NCT03075072) conducted by the Dana-Farber Cancer Institute has further strengthened the case for SRT. This study randomized 196 patients with 5–20 BMs and no prior cranial irradiation to either SRT (intact lesions:20Gy/1fx or 30Gy/5fx; surgical cavities: 25Gy/5fx) or HA-WBRT with memantine (31). The primary endpoint was change in quality of life from baseline to 6 months, assessed by the MD Anderson Symptom Inventory–Brain Tumor (MDASI-BT; score range −10 to 10, with −10 representing best outcome). SRT demonstrated a significant advantage (mean change, −0.32 vs. 0.74; *P* < 0.001). Secondary endpoints also favored SRT, including multiple NCF measures at 12 months (e.g., Hopkins Verbal Learning Test–Revised Total Recall and Delayed Recall, Trail Making Test B). Although SRT was associated with a higher rate of distant intracranial progression (12-month: 45.4% vs 24.2%, *P* = 0.003)—a pattern consistent with earlier SRT vs WBRT studies in oligometastatic disease (2, 3), the authors noted that salvage WBRT was required in only 5.1% of SRS-treated patients within the first year. OS also did not differ between arms (mOS: 8.3 vs 8.5 months, *P* = 0.30) (31). Despite these encouraging results, this Phase III trial had several limitations. Its open-label design may have introduced bias into patient-reported outcomes, the analysis was not stratified by center, and the high early mortality rate (12-month OS: 41.1% for SRT, 42.8% for HA-WBRT with memantine) limited long-term data collection. Thus, further studies are needed to define the optimal role of SRT in this setting (see **Table 1**).

**Table 1:** Summary of ongoing clinical trials comparing SRS/SRT versus WBRT.

Identifier	Phase	Tumor type; planned enrollment	Number of BMs	Intervention vs control	Primary endpoint(s)
NCT01592968	III	Solid Tumors; 88	4-15	SRS vs WBRT	Local Control; Cognitive Function
NCT05807165	II	Solid Tumors; 115	1-4	FSRT vs HA-WBRT + Memantine	IC-PFS; Cognitive Failure-Free Survival
NCT03550391	III	Solid Tumors; 206	5-15	SRS vs HA-WBRT + Memantine	OS; Cognitive Failure-Free Survival
NCT04277403	III	Solid Tumors; 150	4-15	SRS/FSRT vs HA-WBRT + Memantine + SIB	IC-PFS
NCT04804644	III	SCLC; 200	1-10	SRS vs HA-WBRT + Memantine	Time to Cognitive Deterioration
NCT06457906	III	SCLC; 340	1-10	SRS/FSRT vs HA-WBRT + Memantine	Cognitive Function; mOS

SRS, Stereotactic radiosurgery; WBRT,whole brain radiation therapy; HA, Hippocampal avoidance; SCLC,small cell lung carcinoma; IC-PFS,intracranial progression-free survival; SIB,simultaneous integrated boost; BM, Brain Metastasis; OS, overall survival.

### 2.2 SRT for small cell lung cancer brain metastases

Owing to the propensity for multiple brain metastases in SCLC, prophylactic cranial irradiation and WBRT have traditionally been the mainstay of initial management for brain involvement (34). However, OS benefit of PCI is being challenged by advancements in imaging (primarily MRI) and recent clinical studies, for both limited-stage and extensive-stage SCLC (35–38). Despite the development of NCF preservation strategies inspired by NRG CC001 (HA-WBRT + memantine) (4), HA-PCI combined with memantine may still exert a detrimental effect on NCF, particularly in older patients, those with poor performance status, or pre-existing cognitive impairment (39–41).

The incidence of brain metastases at initial SCLC diagnosis is 10-25% (12, 42), rising to 40-50% during the disease course (43). SRS and WBRT are considered when the benefit of PCI is unclear or PCI is not feasible. Several studies have analyzed the efficacy of SRS as a primary local treatment (44–51), summarized in **Table 2**. These investigations suggest that SRS alone yields OS outcomes that are not inferior to WBRT. Studies like the CROSS-FIRE (47) and the Phase II trial NCT03391362 (48) have somewhat challenged the traditional view that SCLC-BM is characterized by exceptionally high neuro-specific mortality and a high risk of widespread intracranial progression.

**Table 2:** Selected studies of SRS for SCLC-BM.

Author/ year	Cases	Intervention/comparison	OS	Intracranial control	Other endpoints	NS
Serizawa T (44); 2002	SCLC: 34 pts NSCLC: 211 pts	SRS: SCLC vs NSCLC	mOS: 9.1 vs 8.6 mo, *P* = 0.43	1-yr LC: 94.5% vs 98%, P = 0.10 mDBC: 6.9 vs 9.8 mo, *P* = 0.44	1-yr QS: 68.9% vs 78.9%, *P* = 0.73	1-yr NS: 86.5% vs 87.9%, *P* = 0.84
Robin, Tyler P (45); 2018	SCLC: PSM SRS: 193 pts WBRT: 1930 pts	SRS vs WBRT	mOS: 10.9 vs 7.6 mo, *P* < 0.001	–	–	–
Rusthoven, Chad G (46); 2020	SCLC: PSM SRS: 187 pts WBRT: 187 pts	SRS vs WBRT	mOS: 6.5 vs 5.2 mo, *P* = 0.003	mTTCP: 9.0 mo vs NA, P < 0.001 mCNS-PFS: 4.0 vs 3.8 mo, *P* = 0.79	–	–
Rusthoven, Chad G (47); 2023	Retrospective Cohort (PSM): 3 groups, 428 pts each Prospective Cohort: 3 groups, 84 pts each	Retrospective: SRS for SCLC vs NSCLC (EGFR/ALK+) vs NSCLC (EGFR/ALK-) Prospective: SRS for SCLC vs NSCLC (TKI+) vs NSCLC (TKI-)	Retrospective Cohort: mOS: 10.4, 23.7, 13.6 mo (Ref: SCLC). NSCLC groups showed significant difference vs SCLC (*P<*0.001; *P* < 0.001). Prospective Cohort: mOS: 8.8, 27.8, 8.9 mo (Ref: SCLC). NSCLC(TKI+): *P* < 0.001; NSCLC(TKI-): *P=*0.48.	Retrospective Cohort 1-yr LBF: 10.2%, 7.0%, 8.0% (Ref: SCLC). NSCLC(EGFR/ALK+): *P=*0.86; NSCLC(EGFR/ALK-): *P=*0.99. 1-yr DBF: 45.9%, 36.4%, 44.8% (Ref: SCLC). NSCLC(EGFR/ALK+): *P=*0.9; NSCLC(EGFR/ALK-): *P=*0.32. Prospective Cohort 1-yr LBF: 9.5%, 4.9%, 4.8% (Ref: SCLC). NSCLC(TKI+): *P=*0.43; NSCLC(TKI-): *P=*0.18. 1-yr DBF: 54.8%, 46.4%, 38.1% (Ref: SCLC). NSCLC(TKI+): *P=*0.36; NSCLC(TKI-): *P=*0.14.	1-yr LMD (non-PSM): Retrospective: SCLC vs NSCLC: 9.7% vs 10.2%, *P=*0.007 Prospective: SCLC vs NSCLC: 10.2% vs 8.1%, *P=*0.82 First Widespread Intracranial Progression#: Retrospective Cohort: 16.9%, 17.2%, 14.4% (Ref: SCLC). NSCLC(EGFR/ALK+): *P>*0.99; NSCLC(EGFR/ALK-): *P=*0.51.	1-yr NS (non-PSM): Retrospective: SCLC vs NSCLC: 90.1% vs 93.5%, *P=*0.83 Prospective: SCLC vs NSCLC: 96.9% vs 96.8%, *P=*0.50
Aizer, Ayal A (48); 2025	SCLC: 100 pts	SRS	mOS: 10.2 mo	1-yr LBF: 15.0% 1-yr DBF: 59.0%	1-yr LMD: 7% 1-yr sRN: 3% Cumulative Salvage WBRT: 22%	1-yr neurologic death: 11.0%*.

SRS, Stereotactic radiosurgery; WBRT, whole brain radiation therapy; SCLC, small cell lung carcinoma; NSCLC, non-small cell lung cancer; pts, patients; EGFR, epidermal growth factor receptor; ALK, anaplastic lymphoma kinase; TKI, tyrosine kinase inhibitors; OS, overall survival; LC, local control; BDC, brain distant control; TTCP, Time to CNS progression; CNS-PFS, central nervous system progression-free survival; LBF, local brain failure; DBF, distant brain failure; ref, reference; QS, qualitative survival; NS, neurological survival; LMD, leptomeningeal metastatic disease; sRN, rsymptomatic adiation necrosis; PSM, propensity score matching; NA, not available; BM, brain metastasis. # Widespread Intracranial Progression: defined as progression of >10 lesions.

* According to the authors, the 1‑year risk of neurologic death with SRS was not only non‑inferior to historical WBRT (one‑sided test, P < 0.01) but also numerically lower (11.0% vs. 17.5%).

Previous reports indicate that NCF decline after WBRT can occur as early as 2 months, whereas SRS demonstrates significant NCF benefits over WBRT (+/- SRS) from 3–4 months onward (49, 50). Given the OS data for SCLC-BM (44–52)and the estimated mOS range (4.0 to 23 months) based on the ds-GPA score (53), at least a subset of patients survives long enough to realize the NCF benefits of SRS (44–48, 52). More durable NCF preservation relies on improved OS from more effective systemic therapies.However, in the immunotherapy era, large Phase III RCTs (e.g.,EXTENTORCH (toripalimab) (54),CAPSTONE-1(adebrelimab) (55), and RATIONALE-312 (tislelizumab) (56)) have shown modest OS benefits from adding first-line immunotherapy to ES-SCLC (54–60).

The current NCCN guidelines list SRS as a treatment option for SCLC-BM (61). The results of ongoing Phase III RCTs (**Table 1**) are eagerly awaited. Additionally, emerging evidence suggests that NCF involves brain structures beyond the hippocampus (62, 63), which presents a challenge to the HA-WBRT/PCI strategy and simultaneously underscores the broader clinical potential of SRT(as will be detailed in the next section).

### 2.3 Connectomics–guided stereotactic radiotherapy: a forward‑looking perspective

The previously observed NCF benefit of SRT over WBRT (+/- HA) is better characterized as a delay in NCF decline—both modalities lead to deterioration from baseline over time (2, 3, 31). Emerging evidence indicates that NCF depends not only on the hippocampus but also on the integrity of key white matter tracts(WMT) that subserve memory, language, executive function, and other cognitive domains (62, 63). This raises the possibility that combining SRT’s dose−painting precision with brain connectomics could further optimize NCF preservation (64).

Brain connectomics refers to the complete set of structural and functional connections within the brain (common networks include the default mode, central executive, salience, dorsal attention, and ventral attention networks). It spans from microscopic axonal connections to macroscopic white matter fiber bundles, the latter being the primary focus in current clinical neuroscience (65, 66). Structural connectivity is typically reconstructed using diffusion MRI and tractography, mapping physical connections between brain regions; functional connectomics relies on resting−state or task−based fMRI to capture cooperative activity across regions (67, 68).

Integrating connectomics into SRT planning may help to deliberately avoid or constrain dose to eloquent white matter bundles, thereby refining treatment plans–an approach that has been reported in benign intracranial diseases (62). In the brain metastasis setting, a single−institution prospective study (reported as a conference abstract in the Red Journal) evaluated this strategy in 84 patients (77 with brain metastases, 182 lesions) (69). Using connectomics to identify 23 key WMT and the hippocampi, dose constraints were applied during SRT planning(single fraction WMT_Dmax_ =12Gy, hippocampi_Dmax_=8.4Gy). With a median follow−up of 28 months, the 12−month local control rate was 98%, and no significant imaging−detected injury to the selected white matter tracts was observed. The mean cognitive impairment rate remained stable (26% at baseline vs 25% at 3 months post−SRT), and mean T−scores for cognitive performance and quality of life showed no significant decline (all *P*>0.1). Importantly, unlike previous studies that reported progressive decline (31), tests of verbal fluency and word retrieval even improved (*P* = 0.02 and *P* = 0.01, respectively). This promising and attractive combination strategy is currently being evaluated in multiple clinical trials (NCT04343157, NCT04073966).

### 2.4 Dose and fractionation optimization in stereotactic radiotherapy

#### 2.4.1 SRS and FSRT

SRS has extensive clinical experience in treating small lesions.The International Stereotactic Radiosurgery Society (ISRS) considers metastases smaller than 1 cm in diameter suitable for safe treatment with Gamma Knife, CyberKnife, or linear accelerator (Linac)-based SRS (70). The recommended SRS dose for intact brain metastases decreases with increasing lesion size(diameter): <2 cm: 24 Gy, 2–3 cm: 18 Gy, 3–4 cm: 15 Gy; or by surgical cavity volume: <4.2 cm^3^: 20 Gy, ≥4.2 to 8.0 cm^3^: 18 Gy, ≥8.0 to 14.4cm^3^: 17 Gy, ≥14.4 to 20.0 cm^3^: 15 Gy, ≥20.0 to 30.0 cm^3^: 14 Gy, ≥30 cm^3^ to <5.0 cm max(diameter): 12 Gy (71, 72) This dose reduction aims to minimize the volume of non-tumor tissue receiving ≥12 Gy (V12Gy), as keeping V12Gy below 8–12 ml is widely accepted to reduce radiation necrosis(RN) risk (73, 74). However, strict adherence to V12Gy constraints may result in a lower biologically effective dose (BED) to the tumor, particularly at the periphery, potentially compromising local control (75).

For larger metastases, FSRT can deliver a higher BED compared to single-fraction SRS (typically 12–18 Gy), offering a radiobiological advantage (76, 77). A comparative study of SRS (18 Gy for 2−3 cm, 15−16 Gy for ≥3 cm) versus FSRT (27 Gy/3 fx) in 289 patients with brain metastases >2 cm found that, after propensity score matching(PSM), FSRT provided superior 1−year local control (91% vs 76%, *P* = 0.01) and lower 1−year RN (8% vs 20%, *P* = 0.01). For lesions ≥3 cm, SRS was associated with a significantly higher RN rate (33% vs 14%, *P* = 0.01) (78). These results align with a meta−analysis of 15 studies involving 1,049 metastases >2 cm, in which FSRT was favored over SRS for both 1−year local control (81.6% vs 69.0%, *P* < 0.0001) and adverse radiation effects(AREs) (8% vs 15.6%, *P* < 0.0001) (76). In the preoperative setting, a study of 404 patients (416 lesions) comparing FSRT (24 Gy/3 fx) and SRS (15 Gy/1 fx) showed that FSRT consistently lowered cavity recurrence after matching (3.3% vs 19.8%, *P* = 0.003), without significant differences in adverse events, leptomeningeal dissemination, or overall survival (79).

Among different FSRT regimens, a dose-response relationship is generally observed (17, 75, 80), supported by studies that have examined this relationship using both the linear-quadratic (LQ) model and the linear-quadratic-cubic (LQC) model—the latter potentially more suitable for the high-dose-per-fraction scenarios typical of FSRT. When calculating BED and fitting tumor control probability models, neither approach reveals an obvious plateau in 1−year local control across a wide range of BED values (approximately 30~70 Gy for the LQ model (80) and 20~60 Gy for the LQC model (75). Consistent with these modeling observations, one retrospective analysis(220pts, 334 intact BMs) showed that total doses of 30~35 Gy in 5 fractions provided better 12-month local control than 22.5~27.5 Gy (33% vs 19%, *P* = 0.03), an effect observed across different tumor types (17). Yet, not all findings align neatly with this dose-response pattern. In the study by Di Perri et al., 273 intact lesions were treated with three FSRT regimens: 30Gy/5fx, 27Gy/3fx, and 35Gy/5fx (BED_10_: 48Gy, 51.3Gy, and 59.5Gy, respectively). The results were discordant: 30Gy/5fx showed higher 12-month local failure than 27Gy/3fx (47% vs 20.4%, *P* = 0.02), yet the 35Gy/5fx regimen (37.5% local failure) did not improve local control over either regimen (*P* = 0.49 and *P* = 0.19). This lack of a clear dose-response likely reflects baseline imbalances: lesions in the 3-fraction group were significantly smaller than those in the 5-fraction groups (median largest diameter 2.0cm vs 2.5cm, *P* < 0.001), and the 27Gy/3fx regimen also delivered a higher BED—both factors may account for its superior local control compared to 30Gy/5fx. Why 35Gy/5fx failed to show added benefit remains unclear; the authors offered no explanation, though unfavorable lesion characteristics or limited sample size may have played a role (81).

The optimal FSRT dose regimen in clinical practice represents a trade-off between maximizing local control and minimizing the risk of RN. In the Di Perri et al. study, for instance, RN risk increased significantly with higher BED, using 30 Gy/5 fx as reference: 27Gy/3fx (HR 3.07, *P* = 0.03) and 35Gy/5fx (HR 4.22, *P* < 0.01) (81). Reflecting such considerations, the ASTRO guideline for brain metastases recommends FSRT regimens of 30Gy/5fx or 27Gy/3fx for intact lesions ≤ 4cm, while for lesions >4 cm, surgery or FSRT is suggested without specifying a particular dose fractionation (72). For the postoperative setting, an ISRS-endorsed systematic review of 13 studies concluded that FSRT/SRT doses in the range of 30~50 Gy (BED_10_) achieve favorable local control, with rates ranging from 60.5% to 91% (median 80.5%) (82).

Despite the aforementioned retrospective data, no compelling evidence from RCTs currently establishes FSRT as equivalent or superior to SRS. Furthermore, the optimal FSRT regimen remains undefined. The final results of several ongoing clinical trials directly comparing FSRT and SRS (see **Table 3**) may help resolve these questions.

**Table 3:** Optimizing stereotactic radiotherapy for brain metastases: a summary of clinical trials.

Identifier	Phase	Planned enrollment	BM characteristics	Intervention vs control	Primary endpoint
SRS vs FSRT					
NCT05703269	III	244	Intact or post-operative lesions (≤15 total), with at least 1 lesion >2 cm or >4 ml; Total GTV ≤30 ml; Excluding LMD	SRS vs FSRT (3–5 fx)	Grade ≥2 Radiation-induced ARE
NCT03697343	III	382	1–10 lesions; Max Diameter: 2–4 cm; Excluding brainstem metastases	SRS vs FSRT (12 fx)	Time to Intracranial Local Progression
NCT05160818	III	126	1–3 post-operative cavities; Max Diameter ≤4 cm; Excluding LMD	SRS vs FSRT (6–7 fx)	ILC
NCT04114981	III	242	Post-operative cavity with max diameter <5 cm. May have concomitant intact lesions (≤3, max diameter ≤4 cm). Excluding brainstem metastases, LMD, or lesions ≤5 mm from optic chiasm.	SRS vs FSRT (3–5 fx)	Cavity Recurrence-Free Survival
NCT06500455	III	269	1–8 intact lesions, Max Diameter: 1–3 cm; May include up to 2 planned-for-resection lesions; Excluding brainstem metastases, LMD, or lesions ≤5 mm from optic chiasm.	SRS vs FSRT (3 fx)	ILC
Pre-operative SRT vs post-operative SRT					
NCT05871307	II	90	≤10 total lesions, with at least 1 planned for resection; Lesion max diameter <5 cm; Lesion located <10 mm from optic chiasm or brainstem; Excluding LMD	Pre-op SRS (within ≤7 days) vs Intra-op SRS vs Post-op SRS (within 2–6 weeks)	Intracranial Treatment Response; ILC
NCT06992973	II	98	Resected lesion ≥3 cm OR symptomatic lesion ≥2 cm but <3 cm; With or without ≤3 small intact lesions	FSRT (27 Gy/3 fx)	ILC
NCT05438212	III	236	Total of 1–4 lesions (max diameter: 2–5 cm); Up to 1 resected lesion; Non-resected lesions ≤4 cm; Excluding brainstem metastases, LMD, or lesions ≤5 mm from optic chiasm.	Pre-op SRS (within ≤7 days) vs Post-op SRS (within ≤30 days)	Time to Composite Adverse Events (Local Progression, Radiation Necrosis, LMD)
NCT03741673	III	180	Lesion ≤4 cm (for SRS) or ≤7 cm (for FSRT); Excluding LMD	Pre-op SRT (within ≤30 days) vs Post-op SRT (within ≤30 days)	1-year LMD-Free Rate
NCT03750227	III	140	≤10 total lesions, with 1 surgical cavity; Non-resected lesions ≤4 cm; Excluding LMD, lesions ≤5 mm from optic chiasm, or lesions >5 cm.	Pre-op SRS (within ≤4 weeks) vs Post-op SRS (within ≤2 weeks)	Time to Composite Adverse Events (Local Progression, Radiation Necrosis, LMD)
NCT05545007	III	146	≤4 total lesions; Lesion max diameter ≥2.1 cm OR <2 cm with mass effect/edema; At least 1 lesion planned for resection.	Pre-op FSRT (within ≤1 week) vs Post-op FSRT (within 4–6 weeks)	Incidence of LMD
Dose escalation/tolerance studies					
NCT02645487	I	135	<10 lesions, each ≤3 cm; Excluding brainstem metastases, LMD	SRS • ≤1 cm (Start 24 Gy): +3 Gy increments, Max 30 Gy • 1–2 cm (Start 21 Gy): +3 Gy increments, Max 27 Gy • 2–3 cm (Start 18 Gy): +3 Gy increments, Max 24 Gy	MTD
NCT02390518	I	50	≤5 lesions; Each lesion ≤4 cm; Excluding brainstem metastases, LMD	SRS • Group 1a (D ≤ 10mm): Start 26 Gy, +2 Gy, Max 30 Gy • Group 1b (11-20mm): Start 26 Gy, +2 Gy, Max 30 Gy • Group 2a (21-30mm): Start 20 Gy, +2 Gy, Max 24 Gy • Group 3 (31-40mm): Start 17 Gy, +2 Gy, Max 23 Gy	MTD
NCT02054689	I	20	≤3 intact lesions; Lesion size 3–5 cm eligible for dose escalation.	FSRT: Start 24 Gy/3 fx; +3 Gy/fx increments, Max 36 Gy/3 fx	Dose-Limiting Toxicity; MTD
NCT03412812	I	13	≤10 lesions	FSRT • 2–4 cm: 35 Gy/5 fx → 40 Gy/5 fx → 45 Gy/5 fx • 4.1-6.0 cm: 30 Gy/5 fx → 35 Gy/5 fx → 40 Gy/5 fx	MTD
Personalized ultra-fractionated adaptive radiotherapy (PULSAR)					
NCT07139990	I	45	≤5 lesions; Each lesion 2–5 cm (Brainstem lesions 1.5–5 cm); Excluding LMD	Pulse 1: Initial FSRT/SRS (3 fx), at least every other day, ≥1 fx/week. Pulse 2: Based on 4-week post-Pulse 1 MRI: (1) If lesion shrinkage ≥25%, omit Pulse 2. (2) Otherwise, proceed with Pulse 2 (2 additional fx, potentially dose-escalated).	Whether good response allows omission of Pulse 2

SRS, Stereotactic radiosurgery; FSRT, fractionated stereotactic radiotherapy; LMD, leptomeningeal metastatic disease; BM, brain metastasis; ARE, Adverse Radiation Effects; MTD, maximum tolerated dose; ILC, intracranial local control.

#### 2.4.2 Maximum tolerated dose

The dose for brain metastasis SRS is primarily referenced from the classic RTOG 90–05 study (reported in 2000) (71). This trial explored the maximum tolerated dose (MTD) based on lesion size, recommending 24 Gy for lesions <2 cm, 18 Gy for 2–3 cm, and 15 Gy for 3–4 cm. These recommendations remain largely in use today, despite some adjustments in subsequent clinical practice (13, 83). A retrospective look at RTOG 90–05 suggests that for lesions <2 cm, the dose-limiting toxicity at 24 Gy was still within the pre-defined acceptable range, and the dose was not escalated further for various reasons, indicating the potential MTD for this group may exceed 24 Gy. Furthermore, RTOG 90–05 included a pre-irradiated population with a median re-irradiation interval of 17 months. According to the Michigan re-irradiation model, organ-at-risk tolerance doses are only reduced by approximately 50% for intervals of 1–3 years (84). Therefore, the tolerable SRT dose for newly diagnosed, treatment-naïve brain metastases is likely higher.

Significant proportion of patients still experience progression after SRT, particularly those with large lesions or radio-resistant histologies (8, 10, 85, 86), warranting further exploration of dose tolerance. One Phase I study enrolled 35 patients with brain metastases deemed suitable for surgical resection to explore dose escalation in the preoperative SRS setting, starting at 18 Gy for 2–3 cm, 15 Gy for 3–4 cm, and 12 Gy for 4–6 cm lesions, with 3 Gy increments per level.Final analysis found the MTD was not reached for the 2–3 cm group, while the MTD was 18 Gy for both the 3–4 cm and 4–6 cm groups (87), exceeding the MTD established in RTOG 90-05.

Regarding FSRT, two early trials explored the MTD for FSRT in both intact and postoperative brain metastases. A Phase I/II study enrolled 48 postoperative patients without prior intracranial radiotherapy, stratifying by cavity size (2-4cm). Dose was escalated from 24 Gy in 3 fractions, with no MTD reached up to 33 Gy. Local control was favorable with 27–30Gy/3fx for 2–3 cm cavities and 27Gy/3fx for 3–4 cm cavities (88). Another Phase I study, NCT02054689 (results not yet published in literature), explored the MTD in 20 patients with intact brain metastases,the MTD was successfully escalated to 36Gy/3fx.

In summary, MTD exploration for brain metastases holds promise for achieving better local control in large lesions and radio-resistant histologies. The eventual publication of ongoing dose escalation trials (see **Table 3**) is anticipated to provide valuable clinical insights.

#### 2.4.3 Strategies for reducing adverse effects (low-dose radiotherapy, staged SRS, personalized ultra-fractionated adaptive radiotherapy)

Modern systemic therapy can achieve considerable intracranial response. When intracranial control is not compromised, individualized low-dose radiotherapy may reduce adverse effects. One study of 102 patients with 688 brain metastases investigated the feasibility of low-dose SRS in the context of modern systemic therapy. Using a median dose of 14 Gy, the 1-year and 2-year local failure rates were only 6% and 12%, respectively, with corresponding AREs rates of just 0.8% and 2%. The study concluded that low-dose SRS is feasible in certain clinical scenarios, such as after prior WBRT or multiple SRT sessions, in the presence of numerous or adjacent small lesions, near critical functional areas, or when balancing local control and neurological function preservation is paramount (86). A tumor control probability model derived from multiple SRT studies suggested that for lesions ≤20 mm (except for MBM), even lower SRT doses (e.g.,18 Gy in a single fraction) can achieve excellent local control (19). Study comparing low-dose and standard-dose pre-operative SRS for brain metastases demonstrated excellent efficacy and safety for both regimens, indicating a need for further research to optimize dose and fractionation (89).

Beyond low-dose strategies, staged SRS (90) is another approach to reduce AREs. It leverages interfraction repair of sublethal damage in normal tissues, and tumor reoxygenation and redistribution to increase radiosensitivity (91), particularly for larger lesions. A single-center retrospective study of medium-sized (2–10 cm³) brain metastases found that, after PSM, two-stage SRS provided better local tumor control and functional outcomes compared to single-session SRS (92). Another study comparing staged SRS vs FSRT found both approaches equally effective for medium (4–10 ml) to large (>10 ml) lesions (93).

Personalized Ultra-fractionated Adaptive Radiotherapy (PULSAR) extends the intervals between high-dose radiation “pulses” to weeks or months, allowing adaptive re-planning based on tumor response and concurrent systemic therapy—an advantage over conventional, condensed FSRT schedules. Preclinical data suggest PULSAR combined with immunotherapy improve local tumor control versus conventional FSRT (94). Dohopolski, M. et al. reported the application of this strategy in brain metastases. with a median follow-up of 1.72 years. mOS was not reached, and the 2-year LF rate was only 8.9%. For patients receiving concurrent systemic therapy, the 2-year LF was 5.5%. The rate of grade ≥3 AREs within 2 years was only 8.7%, and concurrent systemic therapy did not increase AREs (*P* = 0.08), indicating promising efficacy and safety for PULSAR, both alone and combined with systemic therapy (95).Given the favorable early intracranial response with PULSAR (the primary “Triple Threat” regimen achieved a median volume reduction of 38.1% by the final adaptive session for the 2–4 cm lesion group (95)), coupled with literature suggesting that short-term tumor shrinkage ≥20% after SRT predicts excellent local control (96), and the need to reduce toxicity in patients with extended survival, Dohopolski, M.’s team designed the Phase I trial NCT07139990. One cohort of this trial investigates the feasibility of adapting the “Triple Threat” regimen based on treatment response:patients with ≥25% shrinkage after the first three pulses omit the final two fractions; all others complete all five.

### 2.5 Stereotactic radiotherapy equipment and radiation quality

#### 2.5.1 Development of SRT equipment

Commercial SRT equipment includes but is not limited to the Varian (acquired by Siemens) series: TrueBeam, Vital Beam, Edge, Halcyon, Ethos, and Trilogy; the Elekta series: Synergy, Infinity, Versa HD, Axesse, Unity (MRI-guided), and Gamma Knife; the Accuray series: CyberKnife, TOMO and its improved product Radixact; others include MRIdian (MRI-guided) from ViewRay and ZAP-X (with unique radiation shielding technology) from ZAP Surgical (64). Except for Gamma Knife, most SRT platforms are Linac-based (97–99). Regarding dose distribution, differences exist between Gamma Knife and Linac-SRT: the former typically prescribes the dose to the 50% isodose line (e.g., 15Gy delivered to the 50% isodose line results in a maximum intratumoral dose of 30Gy), and due to its focused irradiation characteristics, the target dose distribution is relatively inhomogeneous; the latter, however, achieves better intratumoral dose uniformity (100). The dose heterogeneity of Gamma Knife may also be beneficial, as the resulting dose hotspots improve local control in radioresistant intratumoral regions (101, 102). Linac-SRT can also achieve dose heterogeneity through techniques such as selective boosting and simultaneous integrated boosting (103). A study analyzing brain metastases treated with single-fraction SRS (Gamma Knife or Linac-based) followed by surgical resection found that the core and peripheral regions of brain metastases, as well as different SRS modalities, exhibit distinct genomic characteristics, although the association between these mechanisms and clinical outcomes remains unelucidated (104).

Due to heterogeneity in technical methods, treatment planning, and patient populations across different centers and/or studies, as well as preferences for specific SRT equipment for particular indications among radiotherapy centers, direct comparisons of different SRT devices have historically been challenging (105, 106). Additionally, dosimetric comparisons are limited by multiple factors: energy selection, dose rate, isocenter setup, coplanar or non-coplanar irradiation, beam modulation methods, and planning algorithms (64, 97). In 2016, a benchmarking study initiated by the UK National Health Service designed SRT plans for 2 cases (3 BMs and 7 BMs, respectively) using different SRS devices (Gamma Knife, CyberKnife, Varian-Novalis, Elekta-Synergy, TOMO) and compared dosimetric parameters. Despite certain differences among devices, most could generate comparable radiotherapy plans (105). In the 2023 updated benchmarking study, an additional case with 14 lesions was included in the brain metastasis group, and new accelerator-based SRS devices were incorporated (Gamma Knife Icon, CyberKnife S7, Versa HD and TrueBeam with Brainlab Elements, Edge with HyperArc, Zap-X7). Compared with earlier studies, the new SRS devices appeared to deliver higher-quality treatments: CyberKnife and LINAC-SRT devices showed greater consistency, while low-energy devices produced steeper dose gradients (106). Regarding clinical outcomes, a phase III study comparing single-fraction Gamma Knife and LINAC-based SRS for brain oligometastases found no significant differences in the primary endpoint of RN or secondary outcomes (OS,ILC,IDC) (107).

#### 2.5.2 Particle beam stereotactic radiotherapy

Compared to photon-based radiotherapy, particle therapy (proton, heavy ion) offers unique advantages due to its radiophysical properties (e.g., high LET, Bragg peak) and biological effects (e.g., lower oxygen enhancement ratio, induction of complex DNA damage). These characteristics can potentially translate into superior clinical efficacy and reduced adverse effects (108). Although literature evaluating the efficacy and dosimetric advantages of proton and heavy ion SRS for brain metastases is limited (109–111), particle therapy remains a promising developmental direction. A seminal study by Atkins et al. (2016) reported a large case series of 370 patients with 815 brain metastases (54.9% with prior radiotherapy, 44.6% of those with prior WBRT) treated with proton SRS at a median dose of 18 Gy (RBE). The reported 1-year rates were: LBF 8.5%, DBF 48.2%, OS 51.5%, and RN 3.6%. Despite certain study limitations (single-arm design, use of traditional passive scattering technique rather than the now mainstream pencil beam scanning or even micro/mini-beam techniques (109), and lack of reporting organ-at-risk sparing benefits of protons), the study concluded that proton SRS was well-tolerated and achieved local control comparable to photon SRS (110). Another study, involving 20 patients with multiple brain metastases planned for Gamma Knife SRS, compared original plans with re-optimized Linac-based SRS and proton SRS plans across five parameters: CI, GI, V12Gy, V4.5Gy, and mean brain dose. The proton SRS plans demonstrated superiority in multiple parameters over both the Linac-based and Gamma Knife SRS plans (109). Regarding heavy ion therapy, a study replanned three brain metastasis cases (with 3, 5, and 11 lesions, respectively) originally treated with photon VMAT-SRS, creating particle SRS plans (proton, carbon ion, helium ion) for comparison. The different particle SRS plans were comparable to each other. Compared to photon SRS, particle SRS reduced the V12Gy (widely associated with RN risk) for the case with 11 lesions (111).

In summary, new SRS platforms enable higher quality treatments (e.g., Gamma Knife Icon allows for non-invasive frameless fixation and fractionated SRT). Comparative clinical studies across different platforms have not demonstrated differences in tumor control or AREs. Regarding radiation quality, particle beams offer organ-at-risk sparing advantages over photons but are associated with higher costs and limited accessibility. Furthermore, particle SRS must account for its inherent physical uncertainties (e.g., range, lateral scattering) (112). Therefore, without compromising efficacy, the choice of modality should be individualized, considering patient comfort, financial capacity, and the treating radiotherapy center’s expertise (113).

### 2.6 Integration of stereotactic radiotherapy with surgery and systemic therapy

#### 2.6.1 SRT combined with surgery

RCTs have established that adjuvant SRT following surgical resection of brain metastases improves local control compared to observation alone, and preserves NCF while maintaining local control when compared to WBRT (3, 114)). Due to dynamic changes in the surgical cavity’s shape and volume, and potential shifts in precise localization over time (115, 116), it is generally recommended to deliver SRT within 1–2 weeks post-operatively. Delaying beyond four weeks may compromise local control (82, 117).

However, several challenges exist in the clinical practice of post-operative SRT. These include the potential for iatrogenic seeding along the surgical tract, intraoperative dissemination of tumor cells into the cerebrospinal fluid or leptomeninges, and imprecise target delineation due to an incompletely collapsed cavity, subtotal resection, or post-operative scar formation (64, 118, 119). Consequently, local recurrence at the resection cavity and leptomeningeal dissemination (LMD) are not infrequently reported following post-operative SRT (120–122).

For patients planned for surgery, the “neoadjuvant SRT” approach, where SRT is administered before surgery, offers the advantage of precise target delineation on an intact lesion. The efficacy and safety of this sequence have been validated in retrospective studies. One matched cohort study found preoperative SRS significantly reduced 2-year rates of symptomatic RN (4.9% vs 16.4%, *P* = 0.010) and leptomeningeal dissemination (3.2% vs 16.6%, *P* = 0.010) compared to postoperative SRS, without compromising overall survival or intracranial control (123). These findings are corroborated by the multicenter PROPS-BM study, where preoperative SRS in 242 patients yielded low 2-year rates of leptomeningeal disease (7.6%) and AREs (6.8%) (124). An expanded analysis of 404 patients further confirmed a 2-year leptomeningeal dissemination rate of only 5.8% (125).

The potentially more favorable toxicity profile of pre-operative SRS may be attributed to its dosimetric advantages. A clinical study of 45 patients who received post-operative SRS simulated a pre-operative SRS plan based on their pre-surgical MRI. While the PTV volumes were similar between the simulated pre-op and actual post-op plans, the pre-operative SRS plan reduced the V12Gy of normal brain tissue by approximately 30% (*P* = 0.008), achieved superior conformity (*P* = 0.001), and produced a steeper dose gradient (*P* = 0.0018). These factors may explain the lower incidence of RN associated with the neoadjuvant SRT approach (123). Given the promising potential of neoadjuvant SRT, several clinical trials (see **Table 3**) are underway to rigorously evaluate its indications and benefits.

#### 2.6.2 SRT in combination with systemic therapy

**Figure 3:**
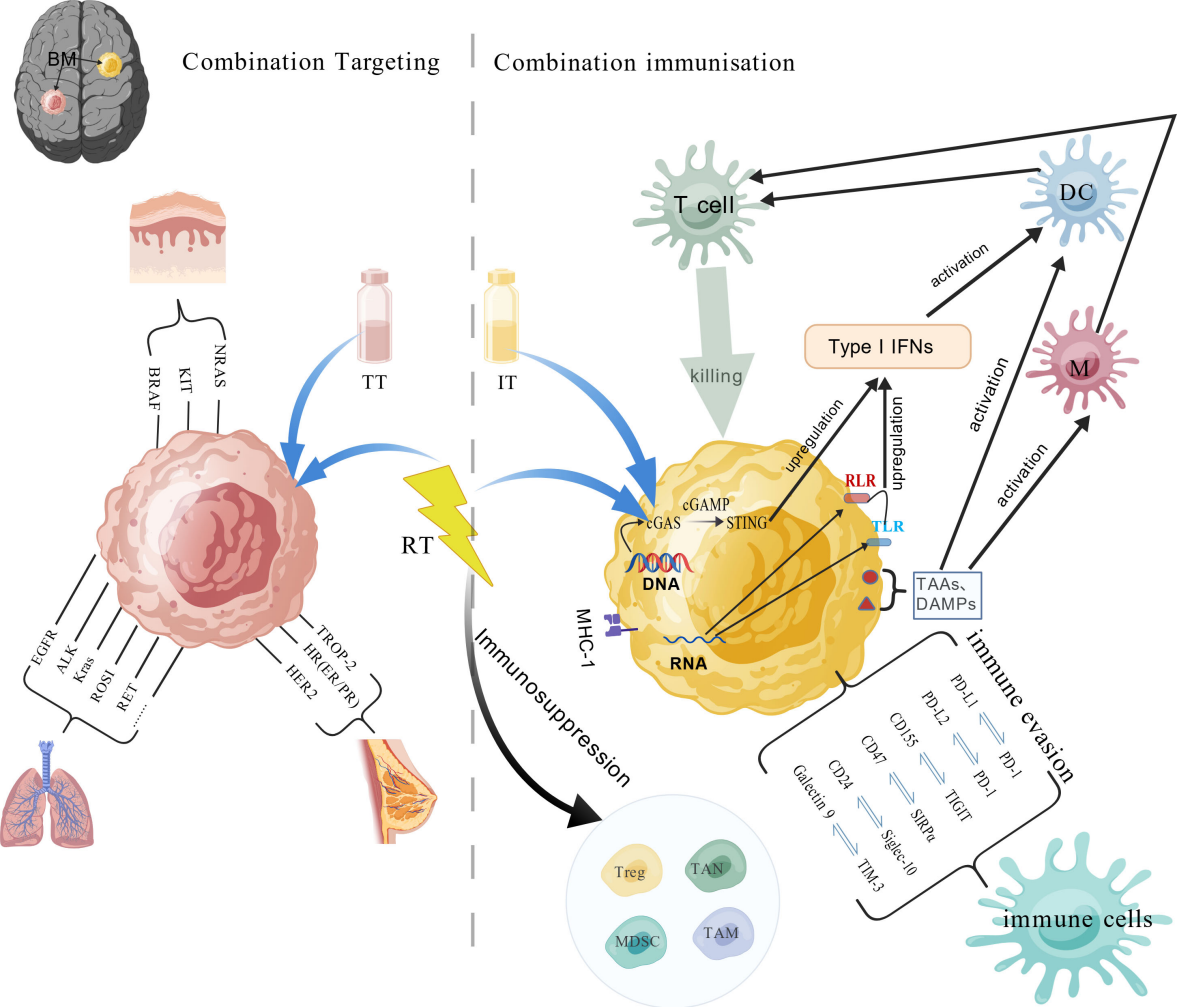
Radiotherapy-Targeted/Immunotherapy Combination Mechanisms. This figure illustrates the common mechanisms underlying the combination of radiotherapy with targeted therapy (left panel) and immunotherapy (right panel). Regarding combination with immunotherapy, the figure also outlines potential mechanisms of radiotherapy-induced immunosuppression. Abbreviations: EGFR, epidermal growth factor receptor; MHC, major histocompatibility complex; ALK, anaplastic lymphoma kinase; NRAS, neuroblastoma RAS viral oncogene homolog; KRAS, Kirsten rat sarcoma viral oncogene homolog; ROS1, ROS proto-oncogene 1, receptor tyrosine kinase; RET, rearranged during transfection; HER2, human epidermal growth factor receptor-2; Treg, regulatory T cell; TAN, tumor-associated neutrophils; TAM, tumor-associated macrophages; MDSC, myeloid-derived suppressor cells; RLR, RIG-I-like receptors; TLR, toll-like receptors; DC, dendritic cells. Created with BioGDP.com. See Jiang, Shuai et al. (2025, DOI: 10.1093/nar/gkae973) for details.

Recent years have witnessed significant advances in systemic therapy for brain metastases. Due to space constraints, this section will focus on the progress and remaining clinical challenges regarding the combination of SRT with systemic therapy in breast cancer, NSCLC, and melanoma (**Figure 3**). For brain metastases with actionable targets (e.g., HER2-positive breast cancer, driver-mutant NSCLC, BRAF-mutant melanoma), agents such as tyrosine kinase inhibitors (TKIs), BRAF inhibitors, and antibody-drug conjugates (ADCs)—are playing an increasingly pivotal role in comprehensive management. For brain metastases lacking clear targets, such as those from triple-negative breast cancer (TNBC), driver-negative NSCLC, and BRAF-wild-type melanoma (and even some BRAF-mutant populations), immunotherapy has also demonstrated promising intracranial activity. Most studies report that combining SRT with systemic therapy improves intracranial control and may even extend OS, with a generally manageable toxicity profile.

#### 2.6.3 Combined with targeted therapy

##### 2.6.3.1 Breast cancer brain metastases

Substantial evidence supports combination therapy for breast cancer brain metastases (BCBM), particularly in HER2-overexpressing subtypes.

###### 2.6.3.1.1 Small molecule TKIs

A retrospective study of 126 patients with HER2+ BCBM, including 47 treated with lapatinib, demonstrated that SRS combined with lapatinib improved intracranial local failure (2-year: 6.5% vs 18.0%, *P* < 0.01) and OS (mOS: 27.3 vs 19.5 months, *P* = 0.03), while exhibiting a lower rate of RN (2-year: 1.9% vs 8.2%, *P* = 0.001) (22). Similarly, study by Miller et al. found that SRT concurrent with TKIs improved 1-year intracranial local failure (5.7% vs 15.1%, *P* < 0.001) and reduced RN (1.3% vs 6.3%, *P* < 0.001) (126). Further supporting this approach, a phase II trial of 40 patients treated with pyrotinib plus capecitabine with radiotherapy(>70% FSRT) reported a 1−year IC−PFS of 74.9% and no symptomatic RN, with mOS not yet reached (23).

###### 2.6.3.1.2 Monoclonal antibodies and ADCs

The study by Miller et al. also reported that SRS concurrent with monoclonal antibodies improved ILC (1-year LF: 10.2% vs 18.4%, *P* = 0.003) (126). In another study of 10 patients with HER2+ BCBM treated with SRS combined with dual-targeted therapy (trastuzumab and pertuzumab) observed no local progression after a median follow-up of 18.3 months, achieved a mOS of 33.9 months, and reported only a single case of asymptomatic RN (127).While earlier ADCs such as T-DM1 were associated with an increased risk of RN when combined with SRT (128, 129), a study of the newer agent trastuzumab deruxtecan (T-DXd) reported a 97% 1-year ILC with only 1% symptomatic RN in 215 treated lesions, supporting its superior intracranial activity and more favorable safety profile (130, 131).

###### 2.6.3.1.3 HR-positive and TNBC brain metastases

Evidence for SRT combined with systemic therapy in HR-positive and triple-negative BCBM is limited. A small retrospective study of 15 patients with 42 HR-positive BCBM lesions treated with SRT and CDK4/6 inhibitors reported an mOS of 36.7 months, a 1-year ILC of 88%, and RN in only 2 lesions (5%) (132). However, Miller et al. found that concurrent endocrine therapy did not improve ILC in HR-positive BCBM (9.6% vs 5.6%, *P* = 0.31) (126). For TNBC-BM, which lacks HER2 and HR targets, radiotherapy combined with TROP-2-directed ADCs (133), PARP inhibitors (134), or anti-angiogenic agents (135) may improve outcomes to some extent, but the evidence remains scarce. Studies exploring SRS combined with targeted agents or immunotherapy (see **Table 4**) are eagerly awaited to provide more robust clinical evidence for these subgroups.

**Table 4:** Summary of selected clinical trials combining SRT with targeted therapy or immunotherapy for brain metastases.

Identifier	Phase	Tumor type; planned enrollment	Brain metastasis characteristics	Intervention vs control	Primary endpoint(s)
SRT combined with targeted therapy					
NCT05553522	I	HER2+ Breast Cancer; 40	≤10 lesions, each ≤3 cm; Excluding LMD or hemorrhagic mets; lesions <5 mm from optic chiasm/nerves	Single-Arm: SRS + Tucatinib (for 2 wks) → Tucatinib + Capecitabine + Trastuzumab until progression/unacceptable toxicity	DLT & Radiotherapy-Related Adverse Events
NCT06088056	II	HER2+ Breast Cancer; 17	New or progressive lesions (≤15); Excluding LMD or hemorrhagic mets	Single-Arm: SRT → T-DXd initiation after a 2-week interval, until progression/unacceptable toxicity	Intracranial ORR
NCT04923542	I/II	HR+ HER2- Breast Cancer; 31	≤15 lesions; Each lesion ≥0.5 cm, largest lesion ≤4 cm; Excluding LMD	Single-Arm: Abemaciclib → SRS after 1-week interval → continue Abemaciclib	IC-PFS
NCT05987644	I/II	ALK+ NSCLC; 56	Asymptomatic or minimally symptomatic lesions, ≤15 lesions	Phase I: Alectinib dose escalation (25 cycles). Phase II Arm A: Alectinib (25 cycles). Phase II Arm B: SRS → Alectinib (within 1 week, for 25 cycles).	Phase I: Safety & Feasibility. Phase II: Composite Endpoint (Intracranial Progression, sRN, Cognitive Decline)
NCT05033691	NA	EGFRm NSCLC; 162	≤20 asymptomatic or minimally symptomatic lesions	Arm 1: Osimertinib → SRT → Osimertinib (consolidation) Arm 2: Osimertinib	IC-PFS
NCT05768490	III	EGFRm NSCLC; 232	≤5 lesions (at least one >5 mm); Lesions >3 mm from optic chiasm/brainstem	Arm 1 (Early SRT): Almonertinib → SRT after 1 month Arm 2 (Delayed SRT): Almonertinib → SRT within 3 months of progression	OS
NCT06248606	II	KRAS G12C-m NSCLC; 30	New or progressive lesions (≤10), largest lesion ≤3 cm; Excluding symptomatic LMD	Single-Arm: Adagrasib + SRS (SRS administered within 3 weeks before or after adagrasib start)	Intracranial Disease Control Rate
SRT combined with immunotherapy					
NCT03449238	I/II	Breast Cancer; 41	2–10 lesions, each ≥0.5 cm, largest lesion ≤4 cm; Excluding lesions <5 mm from optic chiasm/nerves	Single-Arm: SRS → Pembrolizumab initiation after a 4-day interval → Pembrolizumab maintenance until progression/unacceptable toxicity	Abscopal Effect Rate & OS
NCT06238921	I/II	TNBC; 31	Lesions ≥0.5 cm, ≤4 cm; Excluding LMD	Single-Arm: SRS → Sacituzumab Govitecan (SG) + Zimberelimab after 1-week interval → SG + Zimberelimab maintenance	DLT & Progression-Free Survival
NCT04711824	I/II	TNBC or BRCAm Breast Cancer; 41	≤15 lesions, at least one lesion ≥0.5 cm. Excluding LMD or hemorrhagic lesions	Single-Arm: Cycle 1: Olaparib → Concurrent SRS/SRT after 1 week (continue Olaparib until 28 days post-SRT). Subsequent Cycles: Durvalumab + Chemotherapy until progression/unacceptable toxicity.	Adverse Events & Intracranial Disease Control Rate
NCT06839560	II	HER2- Breast Cancer; 102	At least one intracranial lesion ≥10 mm; Excluding leptomeningeal disease	Arm 1: Adebelimab + VEX Chemotherapy + SBRT/SFRT/LDRT (RT & IO separated by 3 weeks, sequence unspecified) Arm 2: Adebelimab + VEX Chemotherapy → Salvage SBRT/SFRT/LDRT upon intracranial progression	IC-PFS
NCT06163820	I/II	Melanoma; 41	Lesions ≥0.5 cm, at least one symptomatic; Excluding lesions with >50% hemorrhage or lesions >5 cm	Single-Arm: 4 cycles Bevacizumab → Nivolumab + Ipilimumab for 4 cycles after 7-day interval (FSRT delivered between Cycle 1 & 2) → Nivolumab maintenance.	Safety
NCT06132945	I	Renal Cell Carcinoma; 20	At least one lesion ≥0.5 cm	SRS/FSRT + Concurrent Cabozantinib + Nivolumab	Treatment Regimen Tolerability
NCT06501391	II	Driver-Negative NSCLC; 54	At least one lesion ≥0.5 cm	Arm 1: ICI + Chemotherapy Arm 2: ICI + Chemotherapy + RT (SRT/WBRT)	Intracranial ORR
NCT06702826	II	NSCLC; 20	Lesion max diameter 1–3 cm; Each lesion volume ≤10 ml, total volume ≤15 ml; Excluding LMD	Single-Arm: SRT + Cadonilimab → Cadonilimab maintenance	Intracranial ORR
Other					
NCT05522660	III	Melanoma or NSCLC; 180	Asymptomatic/minimally symptomatic lesions; ≤10 lesions, at least one ≥0.5 cm, largest lesion ≤30 mm, total volume ≤30 ml	Arm 1: SRS/FSRT (initiated within 2 weeks of randomization) + SOC (Immunotherapy/Targeted Therapy) Arm 2: SOC (Immunotherapy/Targeted Therapy)	IC-PFS

SRS, Stereotactic radiosurgery; NSCLC, non-small cell lung cancer; pts, patients; EGFR, epidermal growth factor receptor; ALK, anaplastic lymphoma kinase; OS, overall survival; IC-PFS, intracranial progression-free survival; LMD, leptomeningeal metastatic disease; sRN, rsymptomatic adiation necrosis; BM, brain metastasis; T-DXd, Trastuzumab-Deruxtecan; KRAS, Kirsten rat sarcoma vial oncogene; SG, Sacituzumab Govitecan; VEX, Vinorelbine+Cyclophosphamide+Capecitabine; ICI, immune checkpoint inhibitors; SOC, standard of care; ORR, objective response rate; DLT, dose-limiting toxicities.

##### 2.6.3.2 Driver-mutant NSCLC brain metastases

Numerous studies have reported on the combination of SRS and targeted therapy for NSCLC-BM. A retrospective multicenter study compared outcomes in EGFR-mutant NSCLC-BM patients receiving SRS followed by TKI, WBRT followed by TKI, or salvage WBRT/SRS upon intracranial progression during initial TKI therapy. Notably, 98% of TKIs used were first-generation erlotinib. A significant difference in OS was observed among the three groups (mOS: 46 vs 30 vs 25 months, *P* < 0.01). Even after PSM, an OS benefit persisted for the upfront radiotherapy approach (HR:0.57, *P* < 0.001), particularly for upfront SRS (HR:0.45, *P* < 0.001) (24).

Given that first-generation TKIs were used in the above study, and third-generation TKIs with superior intracranial efficacy, such as osimertinib (136), are now standard in clinical practice (25), analyzing their combination with radiotherapy is of greater practical relevance. A study in EGFR/ALK-positive NSCLC brain metastases (predominantly treated with osimertinib) found that although adding SRS to TKI therapy did not improve overall survival (*P* = 0.5), it significantly reduced the risk of intracranial progression (HR = 0.63, *P* = 0.033) in multivariable analysis (5).

Several clinical trials investigating SRT combined with targeted therapy are currently underway in driver-positive NSCLC-BM populations, focusing on ALK-positive (NCT05987644), EGFR-positive (NCT05768490), and KRAS-positive (NCT06248606) disease. The results of these studies are anticipated to inform more individualized treatment strategies for brain metastases (see **Table 4**).

##### 2.6.3.3 Melanoma brain metastases

In BRAF-mutant MBM, BRAF inhibitors (BRAFi) and MEK inhibitors (MEKi) exhibit significant intracranial activity (137, 138), with studies supporting their combination with SRS. One retrospective analysis of 24 patients with BRAFm receiving concurrent SRS and vemurafenib(a BRAFi) reported 6- and 12-month ILC of 92% and 75%, respectively, with no added toxicity (139). Another study found that BRAFm patients treated with SRS plus BRAFi had significantly better overall survival (mOS 13.0 vs 1.0/0.5 months, *P* = 0.018) and ILC (92% vs 82.4%/69.2%, *P* = 0.022) compared to those without BRAFi or with wild-type BRAF (140). Dual pathway inhibition (BRAFi + MEKi) may further enhance disease control (7, 141); in a series of 19 metastases treated with SRS plus BRAFi/MEKi, only one local progression occurred by 21.7 months, 1-year lDC was 80%, and mOS reached 20.0 months without additional toxicity (141).

Despite the efficacy of SRS combined with targeted therapy in BRAFm MBM, challenges remain, including the development of resistance to BRAFi, limited duration of response (137, 138, 142), and the emerging evidence favoring immunotherapy (regardless of BRAF status) for superior long-term benefit in some contexts (143, 144). Consequently, SRS combined with immunotherapy has become a viable, and sometimes preferred, alternative strategy for MBM, which will be discussed in the following section on combination with immunotherapy.

#### 2.6.4 Combined with immunotherapy

Radiotherapy activates local and systemic (abscopal) immune responses through multiple, interconnected mechanisms (**Figure 3**): (1) Beyond inducing DNA damage and tumor cell death, irradiation promotes the release of tumor-associated antigens and damage-associated molecular patterns. These signals facilitate the maturation and activation of dendritic cells and macrophages, mediating immunogenic cell death (145). (2) Radiation-induced cytosolic DNA activates the canonical cGAS-STING pathway, leading to type I interferon production. Type I IFN signaling stimulates dendritic cell maturation and enhances adaptive anti-tumor immunity. Alternatively, free RNA released from damaged cells can trigger type I IFN expression via Toll-like receptors (TLRs) and RIG-I-like receptors (RLRs) (146, 147). (3) Radiotherapy modulates the tumor immune microenvironment by upregulating MHC expression and can promote the expression of immune checkpoint molecules(ICIs) (e.g., PD-1, CTLA-4) on lymphocytes, potentially augmenting the efficacy of ICIs (146). These mechanisms, supported by extensive preclinical evidence (148–151), provide a strong rationale for combining SRT with immunotherapy.

##### 2.6.4.1 Breast cancer brain metastases

Genomic analyses indicate that approximately 38.71% of BCBM express PD-L1—a prevalence notably higher across subtypes (27.27-57.14%) than in extracranial disease (12.99-42.86%)—suggesting a rationale for immunotherapy (152). However, pivotal clinical trials demonstrating immunotherapy benefit in advanced TNBC—such as Impassion 130 (Atezolizumab) (153), KEYNOTE-355 (Pembrolizumab) (154), and TORCHLIGHT (Toripalimab) (155)—enrolled few patients with brain metastases, and those included were predominantly asymptomatic or had stable disease.

Emerging data point to more encouraging intracranial activity with novel combinations. The phase II trial NCT04303988, presented at ASCO 2025, reported a CNS objective response rate (CNS-ORR) of 77.1% with adebrelimab (anti-PD-L1), bevacizumab, and platinum chemotherapy in 35 patients with TNBC-BM, including those with neurological symptoms(42.9%). Responses were observed irrespective of PD-L1 status (CPS ≥1: 90.9%; CPS <1: 84.6%) (156), outperforming historical regimens such as utidelone-based combinations (CNS-ORR 42.6-67.6%) (157, 158).

Literature on SRS combined with immunotherapy specifically for BCBM remains limited but promising.A phase I study of SRS plus nivolumab reported a CNS-ORR of 92% and mOS of 24.7 months without RN; intracranial control correlated with early decreases in CD4+ Treg cells (*P* = 0.03)) and later increases in CD4+ effector memory T cells (*P* = 0.011) (159, 160). Another retrospective analysis identified immunotherapy as a favorable predictor for OS (*P* = 0.011) (161). In summary, more clinical research is warranted to establish the role of SRS combined with immunotherapy for BCBM (see **Table 4**).

##### 2.6.4.2 NSCLC brain metastases

A substantial body of literature exists regarding the combination of SRS and immunotherapy for NSCLC-BM. One study demonstrated that SRS delivered concurrently with immunotherapy, compared to SRS alone, improved both OS (mOS: 16.9 vs 12.0 months, *P* = 0.006) and IC-PFS (mIC-PFS: 7.9 vs 5.7 months, *P* = 0.047). The incidence of grade ≥3 adverse events was not significantly different between the two groups (3 vs 5 cases, *P* = 0.75) (162). The benefit of concurrent radiotherapy and immunotherapy for IC-PFS was subsequently corroborated by two prospective studies: the C-brain study (163) and the CTONG2003 study (164).

Given the superior intracranial efficacy demonstrated by dual immune checkpoint inhibition (D-ICPI) in the CheckMate 9LA (165, 166) and CheckMate 227 trials (167), SRS combined with D-ICPI represents another strategic option. A single-arm clinical trial indicated that SRS combined with D-ICPI is tolerable (168). Furthermore, a retrospective study of 288 patients (160 with NSCLC) found that SRS combined with D-ICPI improved ILC compared to SRS alone, with a 1-year local control rate of 94.73% vs 88.26% (*P* = 0.0032) (169).

##### 2.6.4.3 Melanoma brain metastases

Similar to the findings in NSCLC-BM, the CheckMate-204 (170) and Phase II ABC (171) studies demonstrated superior intracranial response with D-ICPI compared to single-agent ICPI (S-ICPI). Evidence for SRS combined with D-ICPI in MBM is substantial (6, 7, 172). An 18−center retrospective study found that, compared to D−ICPI alone, both concurrent and sequential SRS combined with D−ICPI were associated with a reduced risk of death (sequential HR 0.45, concurrent HR 0.48; both *P* < 0.05), with no significant OS difference between the two SRT strategies (172).

Another study of 257 MBM patients reported that D-ICPI plus SRS provided a greater reduction in the risk of death (HR 0.36, *P* < 0.05) and distant intracranial progression (HR 0.47, *P* < 0.05) versus chemotherapy, outperforming BRAFi/MEKi-based combinations (6). The benefit of combined immunotherapy on DIC was further supported by a smaller study of 233 BMs, categorized into SRS+immunotherapy, SRS+targeted therapy, and SRS alone groups. The 1-year DIC were 60% vs 10% vs 11.5%, respectively (*P* < 0.001). Subgroup analysis, as expected, confirmed that SRS+D-ICPI improved the 6-month DIC compared to SRS+S-ICPI (84% vs 50%, *P* = 0.016) (7).

#### 2.6.5 Optimizing the combination of radiotherapy with targeted therapy and immunotherapy

##### 2.6.5.1 Timing and sequencing of combination therapy

Various strategies exist for combining systemic therapy with SRT, each with its own rationale and limitations:

Systemic Therapy Followed by SRT (or Salvage SRT at Progression): To mitigate concerns over concurrent toxicity, guidelines suggest a “de-escalation strategy”—deferring or omitting SRT based on initial systemic response—particularly for patients with driver mutations benefiting from targeted agents. This approach applies mainly to small, asymptomatic, stable metastases (10, 11), aiming to reduce intracranial burden and potentially conver candidates who would otherwise require WBRT into potential SRT recipients, thereby avoiding WBRT-related toxicities. A Phase II trial (NCT06649058) is evaluating the feasibility of this approach by tailoring subsequent therapy based on intracranial response to initial systemic treatment (e.g., MRI surveillance for complete responders vs SRS for those without complete respond). However, the risks and benefits of delaying radiotherapy must be carefully weighed, considering factors such as the interval between therapies, toxicity from systemic agents, and the potential proliferation of resistant clones.

SRT Followed by Systemic Therapy: For symptomatic or active metastases without targetable drivers, upfront SRT remains standard. Strong support comes from the HER2 CLIMB trial(tucatinib vs placebo): in patients with brain metastases, those with disease stable after local therapy derived the greatest IC-PFS benefit (mIC-PFS: 13.9 vs. 5.6 months; HR 0.31, *P* = 0.002) (173), a benefit maintained with longer follow-up (20). This underscores the value of first stabilizing visible disease.Similarly, the pros and cons of delaying systemic therapy must be carefully considered, factoring in the interval before starting drugs and the risk of intracranial progression of occult lesions or extracranial seeding in the absence of systemic control.

Concurrent SRT and Systemic Therapy: This strategy, where SRT addresses visible, localized disease while systemic therapy targets microscopic or small lesions, is increasingly used to achieve high local control while maintaining DIC. However, it requires careful design to balance the risk of increased toxicities. A significant challenge in interpreting the literature is the inconsistent and often broad definition of “concurrent” therapy across studies (e.g., within 2 weeks (172, 174, 175), within 5 drug half-lives (22, 176, 177), or within 4 weeks/1 month (178, 179)), which sometimes blurs the distinction from sequential approaches and hinders cross-trial comparisons.

##### 2.6.5.2 Radiation necrosis

A considerable body of literature indicates that combining SRT with targeted therapy, particularly ADCs, increases the risk of RN. For instance, a meta-analysis reported a notably high rate (17%) of Grade ≥3 RN with the concurrent use of T-DM1 (129). In contrast, newer-generation ADCs appear to be associated with a lower RN risk when combined with SRT. A study of 34 patients with 215 HER2-positive/HER2-low BCBM treated with SRT and T-DXd reported symptomatic RN in only 3 lesions (1%) (131). This promising safety profile warrants further validation in ongoing trials (e.g., NCT06088056, evaluating the efficacy and safety of T-DXd combined with SRT).

The impact of immunotherapy on RN risk remains controversial. While some studies suggest an increased risk with its addition (180), others report conflicting findings (172, 181, 182). An analysis by Lehrer, Eric J. et al. of 203 MBM treated with SRS across 11 centers found that concurrent immunotherapy did not increase the risk of RN. Instead, BRAF mutation status was identified as a risk factor for symptomatic RN (OR: 2.20; *P* = 0.040) (181). A subsequent pan-cancer analysis confirmed RN risk was primarily linked to V12Gy volume, not immunotherapy (182), and another study reported no significant difference in RN between concurrent and sequential SRS-immunotherapy (172). Interestingly, in their analysis of SRS combined with immunotherapy, Lehrer, Eric J. et al. also observed that treatment-related imaging changes (including imaging-defined RN) were predictive of improved OS (HR 0.66, *P* = 0.03) (183). This suggests that such radiological changes may represent a positive biomarker, potentially indicative of enhanced intratumoral immune activation (184).

##### 2.6.5.3 Intratumoral Hemorrhage in Brain Metastases

Intratumoral hemorrhage (ITH) in brain metastases is associated with abnormal proliferation of vascular endothelial cells, intratumoral vascular distortion, and a lack of normal vessel wall hierarchy (185, 186). The incidence of ITH is relatively high in MBM. One earlier study investigating the hemorrhage rates across different intracranial tumor pathologies reported ITH in 50% (7 out of 14) of MBM cases (187).

Combining SRS with BRAFi increases the risk of intracranial hematoma. One study reported a significant increase (10.4% vs 3%, *P* = 0.03) (188). Another study in BRAF-mutant MBM reported a significantly lower 1−year freedom from ITH with SRS plus BRAFi compared to SRS alone (39.3% vs 77.0%, *P* = 0.003) (189). These findings are reinforced by a meta-analysis which confirmed that the combination significantly elevates the risk of intracranial hematoma (OR 3.16, *P* = 0.004) (190). Evidence regarding immunotherapy is less consistent: while one study noted an increased hemorrhage risk with concurrent SRS and immunotherapy (191), a separate meta−analysis found no significant association (192).

##### 2.6.5.4 Hyperprogression following immunotherapy

Hyperprogression (HPD) is typically defined as a ≥2-fold increase in tumor burden within 3 months of initiating immunotherapy. A study investigating patients with NSCLC-BM or MBM who received immunotherapy after neurosurgical resection reported HPD in 5 out of 25 cases (20%). The authors hypothesized that radiotherapy-induced alterations in the tumor microenvironment might contribute to HPD, noting that 4 of the 5 patients with HPD had received prior radiotherapy (3 SRS, 1 WBRT) before immunotherapy (193). The mechanisms underlying hyperprogression after immunotherapy remain incompletely understood. Potential contributors include intrinsic immunological factors, acquired resistance, and external factors such as prior radiation therapy (194).

##### 2.6.5.5 Discordance in biomarker status between primary and brain metastatic tumors

Tumors consist of heterogeneous cell populations, and discordance in biomarker status between primary tumors and their corresponding brain metastases is particularly notable in breast cancer and lung cancer (195–197). A meta-analysis of 15 studies involving 1,373 patients with BCBM found that 42.7% exhibited discordant receptor status between cranial and extracranial sites, predominantly characterized by loss of ER/PR and gain of HER2 expression in brain lesions (195). In lung cancer brain metastases, a meta-analysis of 19 studies comprising 501 cases reported discordance rates for EGFR and KRAS mutations of approximately 10% and 13%, respectively, between primary and brain metastatic sites (196). Another meta-analysis of 6 studies including 230 cases demonstrated a 20% discordance in PD-L1 expression levels between primary lung tumors and their brain metastases (197).

In clinical scenarios where surgical acquisition of brain metastasis tissue for pathological confirmation is not feasible, the impact of selecting targeted therapy or immunotherapy based solely on extracranial biomarker status, when combined with SRS, remains unclear.

##### 2.6.5.6 Reversal of immunosuppression

Radiotherapy exhibits a dual role in modulating the immune system, capable of both activating and suppressing immune responses. It can increase the population of myeloid-derived suppressor cells in both the peripheral blood and the peritumoral area, which promote radioresistance and immunosuppression via pathways such as CCR2 signaling (198, 199). Radiotherapy can also induce phenotypic shifts in tumor-associated neutrophils and tumor-associated macrophages, polarizing them from the anti-tumor N1/M1 phenotypes towards the pro-tumorigenic N2/M2 phenotypes (200). Furthermore, the combination of radiotherapy and immunotherapy can activate immunosuppressive regulatory T cells, which subsequently inhibit effective anti-tumor immunity by impeding T cell activation or being recruited into the tumor microenvironment (145).

The radiation dose and fractionation schedule are also intricately linked to the resulting immune effects. One study found that the priming of CD8+ T cells occurs at fraction doses below 12 Gy. Doses exceeding this threshold can activate the Trex1 enzyme, which degrades cytosolic DNA and consequently prevents STING pathway activation (201). Another preclinical study demonstrated that a single 20 Gy fraction recruited more intratumoral Tregs compared to a regimen of 12 Gy in 3 fractions (202).

Beyond the well-characterized immune checkpoints PD-1/PD-L1 and CTLA-4/CD80/CD86, tumor cells employ other mechanisms for immune evasion. These include alternative pathways such as PD-1/PD-L2, LAG-3/MHC-II or Galectin-3, TIM-3/Galectin-9/HMGB1/CEACAM1, and TIGIT/CD155 (147), which may explain the lack of response to conventional immune checkpoint inhibitors in some patient populations.

In summary, research focusing on optimizing radiotherapy dose/fractionation and developing strategies to counteract specific immunosuppressive mechanisms—such as using anti-CSF1R to block TAM recruitment or anti-Ly6G to deplete tumor-associated neutrophils (145)—is crucial for effectively converting the immunosuppressive tumor response into a potent anti-tumor effect.

## 3 Summary

This review offers a detailed and comprehensive summary of recent advancements in SRT for brain metastases. Key areas discussed include the re-establishment of SRT priority in the context of modern HA-WBRT (combined with memantine), its evolving role in patients with extensive brain metastases and those with SCLC-BM, and the optimization of SRT dose and fractionation. Furthermore, the review discusses how technological advancements in SRT equipment and radiation quality may translate into clinical benefits, such as reduced adverse effects, improved local control, and optimized dosimetric parameters.

Current evidence robustly supports the effective integration of SRT with modern systemic therapies, highlighting its critical role in both pre-operative and post-operative management of brain metastases. However, the review also acknowledges that several aspects of combination therapy require nuanced optimization. These include determining the optimal sequence, managing toxicities like RN and intratumoral hemorrhage, understanding phenomena such as hyperprogression, accounting for discordant biomarker status between primary and metastatic sites, and developing strategies to counteract radiotherapy-induced immunosuppression. In light of these complexities, the importance of a multidisciplinary approach in the management of brain metastases is increasingly paramount.
