# Correction: Stereotactic radiotherapy for brain metastases: indications, dose fractionation, technological innovations, and evolving combination strategies – a comprehensive review

**DOI:** 10.3389/fonc.2026.1885426

**Published:** 2026-06-18

**Authors:** Hu Chen, Shuai Li, Qiuyu Yang, Fangzheng Zhou

**Affiliations:** 1 Department of Radiation Oncology, Shenzhen Luohu Hospital of Traditional Chinese Medicine(Shanghai University of Traditional Chinese Medicine, Shenzhen Hospital), Shenzhen, China; 2 Department of Oncology and Hematology, Shenzhen Luohu Hospital of Traditional Chinese Medicine (Shanghai University of Traditional Chinese Medicine, Shenzhen Hospital), Shenzhen, China; 3 Department of Radiation Oncology, Shenzhen Luohu Hospital Group Luohu People’s Hospital (Third Affiliated Hospital of Shenzhen University), Shenzhen, China

**Keywords:** advance, brain metastases, combined modality therapy, stereotactic radiotherapy, stereotactic radiotherapy equipment

There was a mistake in the caption of **Figure 2** and **Figure 3** as published. “The attribution to BioGDP.com” was omitted from the original captions.

The corrected captions appears below.

“Figure 2 Advances in Stereotactic Radiotherapy for Brain Metastases. This figure outlines the structure of the review and summarizes the advances and optimization strategies in stereotactic radiotherapy for brain metastases. Abbreviations: SRT, stereotactic radiotherapy; SRS, stereotactic radiosurgery; FSRT, fractionated stereotactic radiotherapy; SCLC, small cell lung carcinoma. Created with BioGDP.com. See Jiang, Shuai et al. (2025, DOI: 10.1093/nar/gkae973) for details.”

“Figure 3 Radiotherapy-Targeted/Immunotherapy Combination Mechanisms. This figure illustrates the common mechanisms underlying the combination of radiotherapy with targeted therapy (left panel) and immunotherapy (right panel). Regarding combination with immunotherapy, the figure also outlines potential mechanisms of radiotherapy-induced immunosuppression. Abbreviations: EGFR, epidermal growth factor receptor; MHC, major histocompatibility complex; ALK, anaplastic lymphoma kinase; NRAS, neuroblastoma RAS viral oncogene homolog; KRAS, Kirsten rat sarcoma viral oncogene homolog; ROS1, ROS proto-oncogene 1, receptor tyrosine kinase; RET, rearranged during transfection; HER2, human epidermal growth factor receptor-2; Treg, regulatory T cell; TAN, tumor-associated neutrophils; TAM, tumor-associated macrophages; MDSC, myeloid-derived suppressor cells; RLR, RIG-I-like receptors; TLR, toll-like receptors; DC, dendritic cells. Created with BioGDP.com. See Jiang, Shuai et al. (2025, DOI: 10.1093/nar/gkae973) for details.”

The original version of this article has been updated.

